# Dynamic Response of Model Lipid Membranes to Ultrasonic Radiation Force

**DOI:** 10.1371/journal.pone.0077115

**Published:** 2013-10-23

**Authors:** Martin Loynaz Prieto, Ömer Oralkan, Butrus T. Khuri-Yakub, Merritt C. Maduke

**Affiliations:** 1 Department of Molecular and Cellular Physiology, Stanford University School of Medicine, Stanford, California, United States of America; 2 Edward L. Ginzton Laboratory, Stanford University, Stanford, California, United States of America; University of Sydney, Australia

## Abstract

Low-intensity ultrasound can modulate action potential firing in neurons *in vitro* and *in vivo*. It has been suggested that this effect is mediated by mechanical interactions of ultrasound with neural cell membranes. We investigated whether these proposed interactions could be reproduced for further study in a synthetic lipid bilayer system. We measured the response of protein-free model membranes to low-intensity ultrasound using electrophysiology and laser Doppler vibrometry. We find that ultrasonic radiation force causes oscillation and displacement of lipid membranes, resulting in small (<1%) changes in membrane area and capacitance. Under voltage-clamp, the changes in capacitance manifest as capacitive currents with an exponentially decaying sinusoidal time course. The membrane oscillation can be modeled as a fluid dynamic response to a step change in pressure caused by ultrasonic radiation force, which disrupts the balance of forces between bilayer tension and hydrostatic pressure. We also investigated the origin of the radiation force acting on the bilayer. Part of the radiation force results from the reflection of the ultrasound from the solution/air interface above the bilayer (an effect that is specific to our experimental configuration) but part appears to reflect a direct interaction of ultrasound with the bilayer, related to either acoustic streaming or scattering of sound by the bilayer. Based on these results, we conclude that synthetic lipid bilayers can be used to study the effects of ultrasound on cell membranes and membrane proteins.

## Introduction

Ultrasound has a wide range of effects on biological tissue, many of which are not fully understood. In particular, low-intensity ultrasound has a number of effects that are apparently mechanical in nature – as opposed to the predominantly thermal effects of high-intensity ultrasound – but the exact mechanisms involved are not clear. One example of this, and the motivation for the present work, is the modulation of neural activity with low-intensity ultrasound [1], [2], [3]. Although research on the effects of ultrasound on excitable tissue dates back to the 1920s [4], several recent results have revived interest in this phenomenon. Reversible changes in action potential frequency in response to low-intensity ultrasound (on the order of 0.1–10 W/cm^2^) have been observed *in vitro* [5], [6] and *in vivo* [7], [8], [9], [10], [11]. In addition, transducer arrays that transmit ultrasound through the human skull have been implemented in high-intensity focused ultrasound surgery [12], [13], [14], [15], and it has been proposed that this technology could be adapted to deliver low-intensity ultrasound for the treatment of neurological disorders [5], [10].

Modulation of neural activity by ultrasound can occur without significant changes in temperature [10], [11], implicating mechanical effects of ultrasound in this phenomenon. Beyond identifying the rather broad category of mechanical effects, however, definitive evidence for a specific mechanism underlying the neuromodulatory effects of ultrasound has been elusive. One possibility is that the effects of low-intensity ultrasound on neural signaling are mediated by mechanical forces acting on neural cell membranes [10], [11]. This intriguing idea motivated us to investigate whether synthetic lipid bilayers could provide a useful model system for studying mechanical effects of ultrasound on cell membranes. In addition to the neuromodulatory effects of ultrasound, the mechanical interaction of ultrasound with cell membranes is relevant to numerous other biomedical applications of ultrasound. Practically every child born in the developed world has been exposed to ultrasound *in utero*, and diagnostic and therapeutic ultrasound procedures are routinely used, despite our incomplete understanding of ultrasound’s effects on biological tissue, and even on the simplest components of biological systems.

Previous work suggested that synthetic lipid bilayers do not respond to low-intensity ultrasound [16], [17], [18], a result that is apparently inconsistent with the proposed mechanical basis for the neuromodulatory effects of ultrasound. In light of the renewed interest in ultrasonic neuromodulation and the continuing use of other biomedical ultrasound technologies, it therefore seemed worthwhile to revisit the effects of ultrasound on synthetic lipid bilayers.

Mechanical effects of ultrasound can be classified as due to either *cavitation* or *radiation force*. These two classes of mechanical effect differ greatly with respect to the time scale of the forces involved. Cavitation is a response to the changes in pressure and density that occur at the acoustic frequency, resulting in the expansion, oscillation, and collapse of tiny gas bodies in the tissue or solution [19], [20]. Radiation force is a mechanical body force caused by spatial gradients in acoustic intensity (for example, due to absorption or reflection of sound) [21], [22], [23], [24]. Thus, cavitation is a response to the first-order, oscillating stresses associated with the acoustic pressure, whereas radiation force is a constant force and a second-order effect of the ultrasound field. Differentiating between these two possible sources of mechanical effects is critical in understanding effects of low-intensity ultrasound.

We investigated the mechanical effects of low-intensity ultrasound on lipid bilayers using electrophysiology and optical vibrometry. We find that the response of these model membranes to ultrasound can be explained in terms of a response to radiation force, rather than cavitation. Our results indicate that radiation force causes small displacements of the bilayer, changing bilayer curvature and area, and therefore changing bilayer capacitance. The dynamic response of the bilayer to ultrasound can be described in terms of an interfacial wave, driven by the interaction between the internal forces due to bilayer tension and the external hydrostatic pressure and radiation force. Our findings support the idea that processes sensitive to the organization of biological membranes can be modulated by radiation force using low-intensity ultrasound, and also highlight the potential importance of membrane dynamics in the biological response to ultrasound. We conclude that synthetic lipid bilayers can be a useful model system for understanding the effects of low-intensity ultrasound on the brain and other complex biological systems.

## Results

### On/Off Currents in Response to Ultrasonic Radiation Force

**Figure 1:**
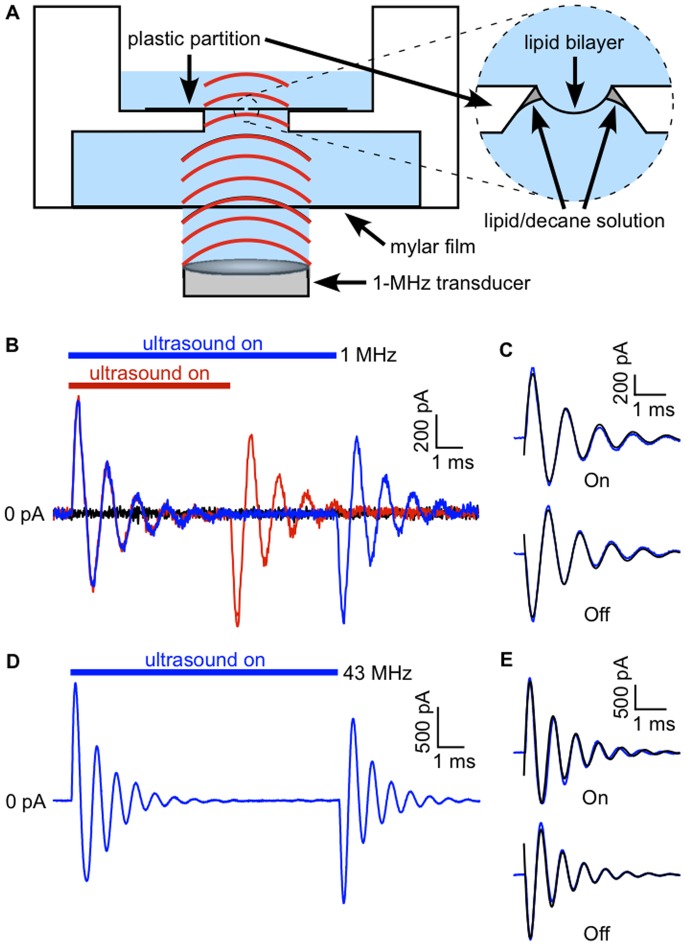
On/Off currents in response to ultrasound in lipid bilayers under voltage-clamp. **A.** Diagram of experimental apparatus. Above the transducer are (from bottom to top): a column of distilled water (∼4 mm) held in place by surface tension, a thin mylar film (0.1 mm), a layer of buffered salt solution (5 mm), the lipid bilayer (∼4 nm thick; on the order of 0.1 mm in diameter) and its supporting partition (0.2 mm), and another layer of buffered salt solution (1.7 mm). **B.** Currents in response to 10-ms (*blue*) and 6-ms (*red*) ultrasound applications at 1 MHz and 610 mW/cm^2^ (estimated power intensity), along with the baseline current in the absence of ultrasound stimulation (*black*) in a bilayer voltage-clamped at −200 mV. The bilayer was formed from the lipids POPE and POPG (3∶1 by weight). The capacitance of the bilayer was 170 pF. **C.** Fits of Eq. 1 (*black lines*) to the On and Off components of the current in ***A***, but showing the average of 20 ultrasound applications. Values of the fit parameters (± SD) are *a*, 800±7 pA; *f*, 900±1 Hz; *α*, 700±9 s^−1^; *φ*, −0.21±0.01 radians for the On response and *a*, −790±7 pA; *f*, 950±1 Hz; *α*, 720±9 s^−1^; *φ*, −0.25±0.01 radians for the Off response. **D.** Currents in response to a 10-ms ultrasound application using a focused transducer (∼90 µm focal spot size) at 43 MHz and 5 W/cm^2^ in another POPE/POPG (3∶1) bilayer, voltage-clamped at −200 mV. The current trace is the average of 16 ultrasound applications. The capacitance of the bilayer was 170 pF. **E.** Fits of Eq. 1 (*black lines*) to the On and Off components of the current in ***C***. Values of the fit parameters (± SD) are *a*, 1700±15 pA; *f*, 1300±1 Hz; *α*, 880±10 s^−1^; *φ*, −0.27±0.01 radians for the On response and *a*, −1600±11 pA; *f*, 1400±1 Hz; *α*, 1000±9 s^−1^; *φ*, −0.04±0.01 radians for the Off response.

Lipid bilayers under voltage-clamp display a robust electrical current response to ultrasound, characterized by On/Off behavior and damped oscillations (**Fig. 1**). We first investigated the effects of ultrasound at 1 MHz. The response of a bilayer, voltage-clamped at −200 mV, to a 10-ms ultrasound application is shown in blue, along with the response to a 6-ms application in red (**Fig. 1** ***B***). At the onset of the ultrasound stimulus a sinusoidal current oscillation appears. The amplitude of the oscillation decays to <5% of the initial value after approximately 6 ms. A second current oscillation, opposite in polarity but otherwise identical to that at the start of the application, occurs on termination of the stimulus. The response to the 6-ms ultrasound application is identical to that of the 10-ms application except that the start of the second current oscillation is shifted in time to coincide with the end of the stimulus. Thus the current elicited by ultrasound applications longer than the exponential decay time of the oscillations has distinct “On” and “Off” components. The time course of the On and Off current responses can be described by a simple exponentially decaying sine wave function (**Fig. 1** ***C***),
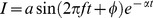
(1)where *a*, *f*, and *φ* are the amplitude, frequency, and phase of the current oscillation, *α* is the damping constant, and the time *t* is relative to the onset or offset of the ultrasound stimulus. This expression gives us numerical parameters with which to compare the response to ultrasound under different experimental conditions.

To determine whether the current response is due to radiation force or cavitation, we stimulated the bilayer with a focused 43-MHz transducer (**Fig. 1** ***D*** **and** ***E***). The response at this higher frequency is similar to that obtained with the 1-MHz transducer, indicating that the electrical response is due to radiation force, rather than cavitation. Since cavitation is a direct response to acoustic pressure, it occurs in a narrow frequency bandwidth with the efficiency dropping off sharply above a resonant frequency (typically on the order of 0.1 to 1 MHz) determined by the size of the oscillating gas body [19]. The wide bandwidth of the response demonstrated here is instead characteristic of radiation force, which to a first approximation is independent of frequency [21].

**Figure 2:**
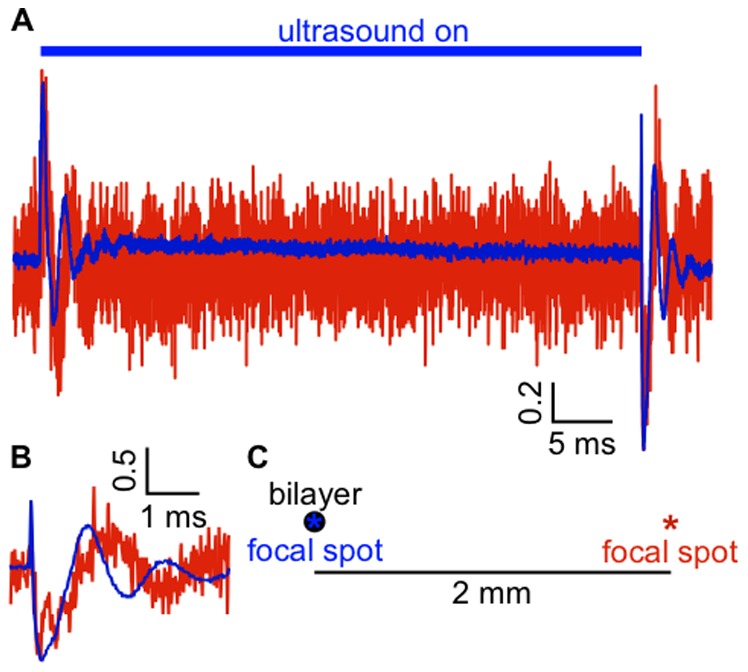
Maximal current response requires alignment of the bilayer and the ultrasound beam. **A.** Current recorded from a POPE/POPG bilayer under voltage-clamp at −100 mV in response to a 50-ms, 13-W/cm^2^ ultrasound pulse from a 43-MHz focused transducer with the focal spot aligned on the bilayer (*blue current trace*) and with the focal spot moved 2 mm away from the bilayer in the *x*-*y* plane (*red current trace*). The currents are normalized to the peak inward current and the baseline currents have been subtracted. The current scale bar is in normalized units. The peak positive and negative currents were 147 and −157 pA with the focal spot centered on the bilayer, and 15 and −13.7 with the focal spot translated 2 mm in the *x*-*y* plane. The capacitance of the bilayer was 153 pF. The currents are the average of 10 ultrasound applications. Mean peak positive and negative currents, relative to the peak current with the bilayer and ultrasound beam aligned, for the On and Off responses (respectively) after moving the transducer were 8.9±0.1% and 9.1±0.1% (mean ± SE, *n* = 7). **B.** The Off current response from **A.** on an expanded time scale. **C.** Approximate scale drawing indicating the position of the focal spot for the two current traces.

The ultrasound beam from the 1-MHz transducer is wider than the bilayer (∼4 mm), so it could be argued that the response is due to radiation force acting on the plastic partition supporting the bilayer, rather than on the bilayer itself. In contrast, the diameter of the 43-MHz transducer at the focal spot is ∼90 µm, so that the beam passes through the aperture on which the bilayer is formed without contacting the partition. When this focal spot was moved 2 mm away from the bilayer in the *x*-*y* plane, the peak outward and inward currents for the On and Off components of the response were less than 10% of the peak currents when the focal spot was centered on the bilayer (**Fig. 2**). We conclude that the response is primarily due to the effect of radiation force on the bilayer itself.

### The Ultrasound-induced Currents Reflect Changes in Bilayer Capacitance

**Figure 3:**
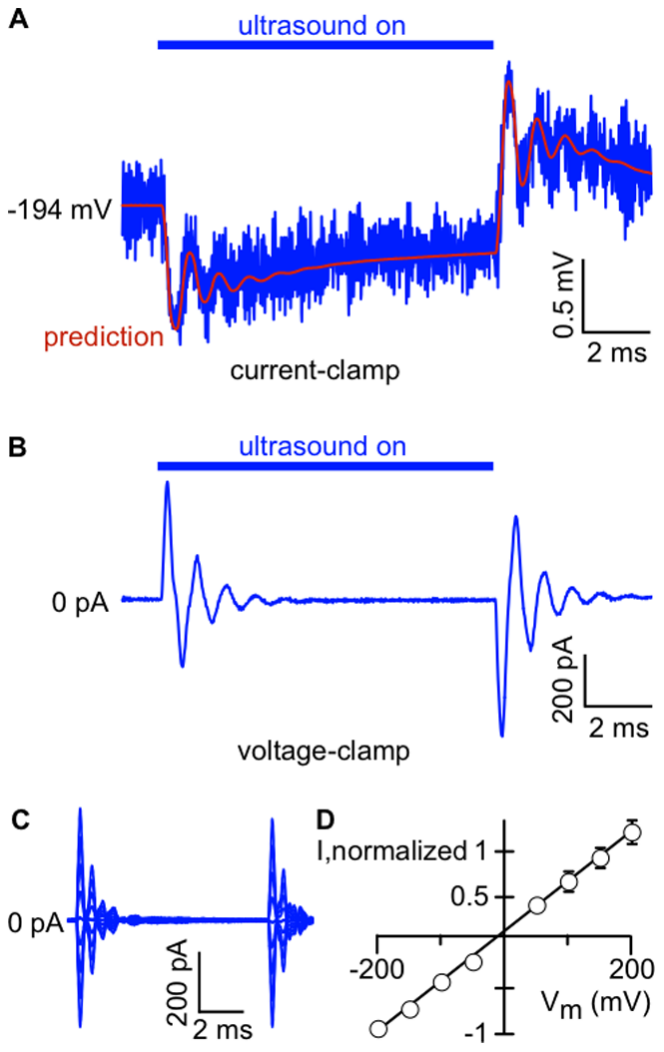
Voltage changes in response to ultrasound are caused by changes in bilayer capacitance. **A.**Voltage changes recorded under “current-clamp”. A 10-ms ultrasound pulse at 1 MHz and 610 mW/cm^2^ was applied to a POPE/POPG (3∶1) bilayer charged to −194 mV (*blue line*). The red line shows the voltage changes predicted from the current recorded in voltage-clamp mode (panel ***B***), calculated using Eq. 3. The voltage trace is the average of 20 ultrasound applications. The capacitance of the bilayer was 110 pF. **B.** Current recorded from the bilayer in ***A*** under voltage-clamp at −200 mV, in response to the same ultrasound stimulus as in ***A***. The current trace is the average of 20 ultrasound applications. **C.** Currents in response to a 10-ms, 1-MHz, 610-mW/cm^2^ ultrasound application in a POPE/POPG (3∶1) bilayer voltage-clamped at potentials from −200 mV to +200 mV, in 50-mV steps. Current traces are the average of 20 ultrasound applications. The capacitance of the bilayer was 130 pF. **D.** Mean (± SE) peak negative or positive current following the end of the ultrasound stimulus, as a function of bilayer voltage, normalized to the peak negative current at −200 mV (*n* = 5). Some error bars are smaller than the symbol size. The black line is a linear fit with slope 5.5±0.1 mV^−1^ and *y*-intercept 0.05±0.01 (± SD).

We hypothesized that the currents in response to ultrasound are capacitive currents (*I_C_*) due to changes in bilayer capacitance (*C*), according to:
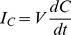
(2)where *V* is the bilayer voltage. Since the currents have both positive and negative components at negative voltage, they are clearly not caused by changes in bilayer resistance (for resistive currents, *I_R_ = V/R*, where *R* is the resistance, giving only currents with the same sign as the voltage). To test the hypothesis that the ultrasound-induced currents are capacitive currents, we monitored changes in bilayer voltage in response to ultrasound using the “current-clamp” setting of the amplifier (**Fig. 3** ***A***). Because the bilayer resistance is very high (>10 GΩ), the amount of charge stored in the bilayer capacitance is essentially constant during the ultrasound stimulus. Under this constant-charge condition, the relationship between the capacitive current in voltage-clamp mode and the voltage changes in current-clamp mode can be approximated for small changes in capacitance by:
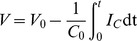
(3)where *V_0_* and *C_0_* are the baseline values of the voltage and capacitance. Thus, if the currents recorded under voltage-clamp are due to changes in capacitance, they can be used to predict the voltage changes measured under current-clamp. We therefore compared the response to ultrasound in the same bilayer in both current-clamp and voltage-clamp modes. We found that the voltage changes in response to ultrasound under current-clamp (**Fig. 3** ***A***) are exactly as predicted by the currents in response to ultrasound under voltage-clamp (**Fig. 3** ***B***), confirming that ultrasound induces changes in bilayer capacitance. In further support of this hypothesis, the current responses under voltage-clamp are linearly dependent on voltage, as expected for a current due to capacitance changes (**Fig. 3** C and D).

### The Capacitance Changes are due to Changes in Bilayer Area

A mechanistic explanation for the capacitance changes in response to ultrasound is that radiation force acting on the bilayer causes changes in bilayer shape, thereby changing its capacitance according to:
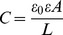
(4)where *A* is the area of the bilayer, *L* is the thickness of its hydrophobic core, *ε_o_* is the permittivity of free space, and *ε* is the relative dielectric constant of the hydrophobic core (∼2). Integrating the capacitive currents in response to ultrasound and dividing by the voltage (as per Eq. 2) indicates that there is a net decrease in capacitance during the ultrasound stimulus (−0.24±0.03% (mean ± SE), *n* = 59, capacitance range 17–3100 pF). Assuming that ultrasound does not change *ε*, this result indicates either a decrease in bilayer area, an increase in bilayer hydrophobic thickness, or both, in response to ultrasound.

**Figure 4:**
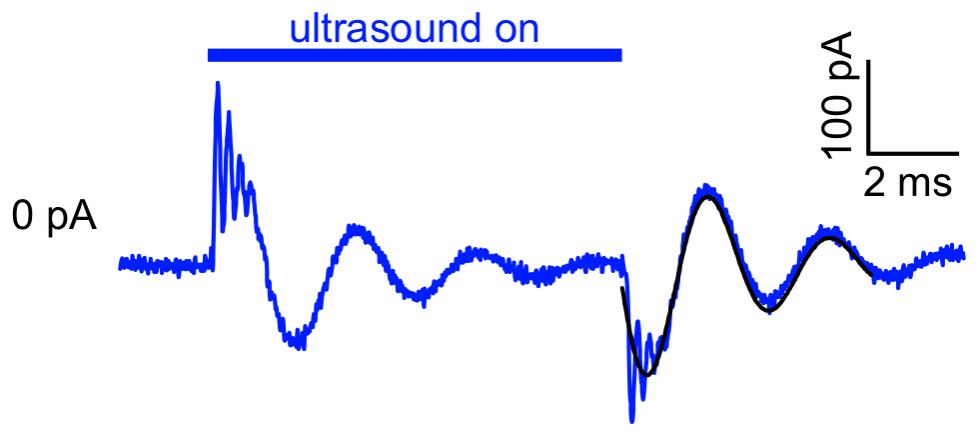
Ultrasound-induced capacitive currents in solvent-free synthetic bilayers. Currents in response to a 10-ms, 1-MHz, 610-mW/cm^2^ ultrasound application in a bilayer formed from a solution of POPE and POPG (3∶1) in squalene (8.3 mg/mL), under voltage-clamp at −200 mV, along with a fit of Eq. 1 to the Off component of the current. For the example shown here, the fit parameters (±SD) were: *a*, −140±3 pA; *f*, 340±1 Hz; *α*, 300±10 s^−1^; *φ*, 0.17±0.02 radians, and the capacitance was 500 pF. The mean (±SE) values were *a*, −110±30 pA; *f*, 400±30 Hz; *α*, 340±50 s^−1^; *φ*, 0.19±0.07 radians, and the capacitance ranged from 80 to 560 pF (*n = *7).

Since the bilayers in our experiments are formed from decane solutions (see **Materials and Methods**), they are surrounded by a torus of lipid/decane solution (**Fig. 1** **** ***A***, *inset*) and contain some decane molecules inserted within the two lipid monolayers [29], so one potential explanation for the decrease in capacitance is that additional decane enters the hydrophobic core of the bilayer from the surrounding torus, thereby increasing the hydrophobic thickness. This additional solvent could take the form of bulk flow of solvent or the migration of discrete “solvent lenses” (pockets of solvent floating in the core of the bilayer). However, we obtained qualitatively similar capacitive current responses in bilayers formed from squalene solutions (**Fig. 4**), despite the fact that squalene cannot enter the hydrophobic core of the bilayer [30]. This result indicates that increasing bilayer thickness caused by movement of solvent molecules between the solvent border and the bilayer is not responsible for the capacitance changes in response to ultrasound.

**Figure 5:**
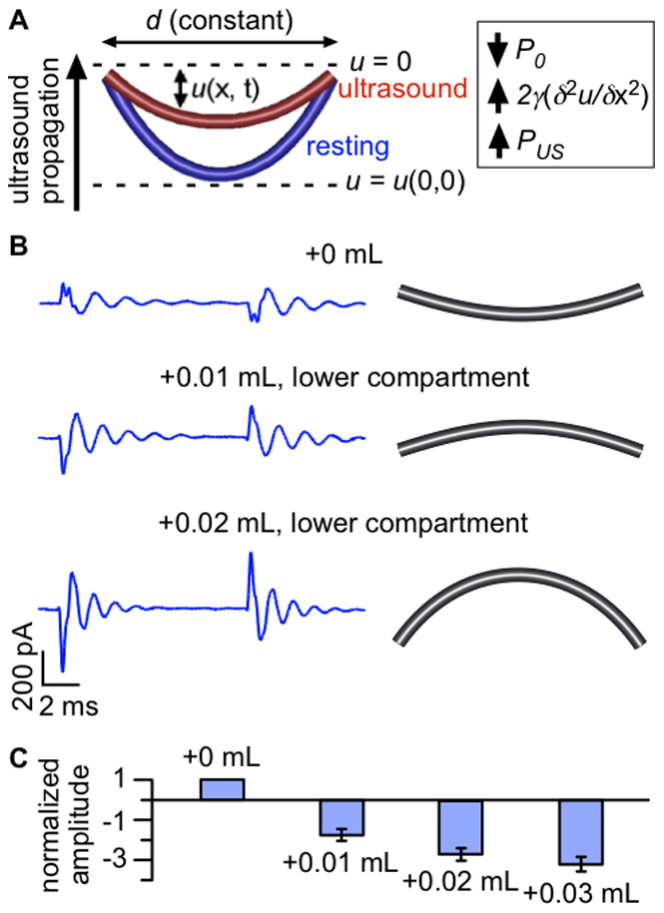
The response to ultrasound depends on bilayer curvature. **A.** Diagram illustrating changes in bilayer shape in response to ultrasound. The bilayer is a curved surface (shown in cross-section) with base of diameter *d*. The bilayer displacement *u*(x, t) is measured relative to the plane *u* = 0, with positive displacement defined to be in the direction of ultrasound propagation. Under standard experimental conditions, the bilayer is curved opposite the direction of ultrasound propagation in the resting state (*blue*). In response to ultrasonic radiation force, bilayer curvature is decreased (radius of curvature increased) and bilayer area is decreased (*red*). The inset summarizes the three types of pressure that determine the bilayer curvature at steady-state: the hydrostatic pressure, *P_0_* (negative); the pressure due to bilayer tension, 2*γ*(*δ^2^u/δ*x^2^) (positive); and the pressure due to radiation force, *P_US_* (positive). The changes in response to ultrasound are exaggerated for the purpose of illustration. **B.** Capacitive currents at −200 mV in response to a 1-MHz, 610-mW/cm^2^ ultrasound pulse with different solution volumes in the lower compartment of the bilayer chamber (*left*), along with illustrations of the inferred resting-state bilayer curvature (*right*). The lower compartment volumes are 0.45 mL (+0 mL, *top*), 0.46 mL (+0.01 mL, *middle*), and 0.47 mL (+0.02 mL, *bottom*). Currents are the average of 10 ultrasound applications and the baseline currents are subtracted. Bilayer capacitance was 170 pF. **C.** Mean (±SE) amplitudes for fits of Eq. 1 to the Off capacitive current at −200 mV in response to ultrasound at 1 MHz and 610 mW/cm^2^ with different solution volumes in the lower compartment of the bilayer chamber, normalized to the amplitude at the initial volume and to bilayer capacitance (see **Materials and** **Methods**, *“Data Analysis”* section) (*n* = 11–15).

A second explanation for the decrease in capacitance is that bilayer area decreases. One might expect the area of the bilayer to expand in response to radiation force, rather than decrease as seen here. However, there is a hydrostatic pressure gradient across the bilayer under our standard experimental conditions, such that the bilayer in its resting state is curved toward the lower compartment of the bilayer chamber, opposite the direction of ultrasound propagation (**Fig. 5** ***A***). We hypothesized that this inward curvature accounts for the decrease in area in response to ultrasound: in this model, the radiation force offsets some of the hydrostatic pressure, causing the bilayer to decrease its curvature, thereby decreasing its area (**Fig. 5** ***A***). According to this hypothesis, it should be possible to reverse the effect of ultrasound – obtaining a net increase in capacitance – by reversing the initial curvature of the bilayer. To test this hypothesis, we manipulated the initial curvature of the bilayer by changing the volume of solution in the lower compartment of the bilayer chamber (thereby changing the hydrostatic pressure on the bilayer) and then examined the effects on the capacitive current. As predicted, we could switch the polarity of the On/Off capacitive current such that the On response was initially negative and the Off response was initially positive at −200 mV (**Fig. 5** ***B***). Adding additional solution after the switch in response polarity increased the amplitude of the response (**Fig. 5** ***B*** **and** ***C***). It is possible that, in addition to affecting bilayer curvature, hydrostatic pressure also affects bilayer tension. However, theoretical considerations [31] suggest that changing hydrostatic pressure cannot change the tension of a bilayer in equilibrium with a border of lipid solution, because the bilayer can change curvature by incorporating material from the border (see below). This conclusion is somewhat controversial [32], [33], but, in any case, changes in bilayer tension cannot explain the change in the polarity of the capacitive current response. Thus, these results produce a clear picture of the bilayer changing in curvature and area in response to ultrasound.

**Figure 6:**
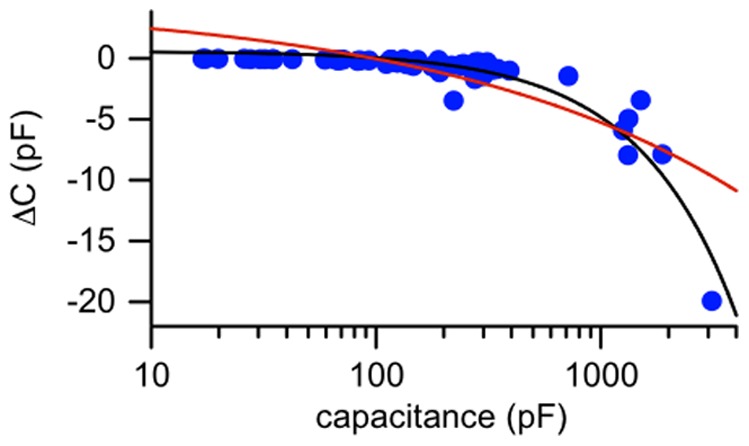
Changes in capacitance in response to ultrasound are proportional to bilayer capacitance. Net (steady-state) change in capacitance as a function of resting bilayer capacitance (logarithmic scale) (*n* = 59). The black line is a linear fit to the net change in capacitance as a function of resting capacitance with a slope of −0.005.4±0.002 pF/pF and *y*-intercept of 0.48±0.15 pF (± SD). The red line is an alternative fit in which the change in capacitance is assumed to be linearly proportional to the bilayer perimeter. Specifically, the fit function is *ΔC* = a*p[*C*]+*b*, where p[*C*] is a function which converts the bilayer capacitance (*C*) to an estimate of the bilayer perimeter (see **Materials and** **Methods**, *“Data Analysis”* section). The values of the fit parameters are *a* = −11.3±1.1 pF/mm, and *b* = 4±0.6 pF (± SD). R^2^ values for the fits are 0.89 for the fit in which capacitance change is linearly proportional to capacitance and 0.64 for the fit in which capacitance change is linearly proportional to perimeter.

There are two mechanisms by which a bilayer surrounded by a torus of lipid/hydrocarbon solution can change its area. Material can be exchanged between the bilayer proper and the surrounding bulk solution, thereby changing the bilayer diameter and the volume of material incorporated into the bilayer; or the bilayer can undergo an elastic area expansion or compression, without changing the bilayer diameter or volume. Because elastic changes in bilayer area occur at constant volume, they are accompanied by a reciprocal change in bilayer thickness according to:
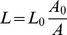
(5)where *L*_0_ and *A*_0_ are the resting values of the thickness and area, and a change in the area per lipid molecule. Only the elastic mechanism is physiologically relevant. Two arguments suggest that an elastic area change, rather than exchange of material with the bulk solution, is involved in our experiments. First, exchange with the bulk solution changes bilayer diameter and should therefore result in a capacitance change linearly proportional to the bilayer perimeter, while for an elastic area expansion or compression the change should be linearly proportional to the bilayer area (and therefore linearly proportional to bilayer capacitance) [34]. We find that the capacitance change in response to ultrasound is proportional to bilayer capacitance rather than bilayer perimeter (**Fig. 6**). Second, previous work indicates that exchange of lipid material between the bilayer and solvent border is a slow process relative to the frequencies of the capacitive current response and indeed to the duration of the ultrasound applications in our experiments [28], [35], [36], [37]. In other words, the time required for the bilayer to equilibrate with the solvent border is much longer than the relevant time scales in our experiments. In particular, in studies that separated the contributions of these two types of area changes, it was found that capacitance changes are dominated by the elastic mechanism at frequencies above 1 Hz [28], [36], [37]. In this context, we note that the time course of the pressure change associated with the ultrasonic radiation force is essentially a step function (see **Fig. S1** and the text in **File S1**). We therefore conclude that the bilayer area changes we observe are primarily due to elastic expansion and compression at constant volume.

### Fluid Dynamic Model of the Response to Ultrasound

**Figure 7:**
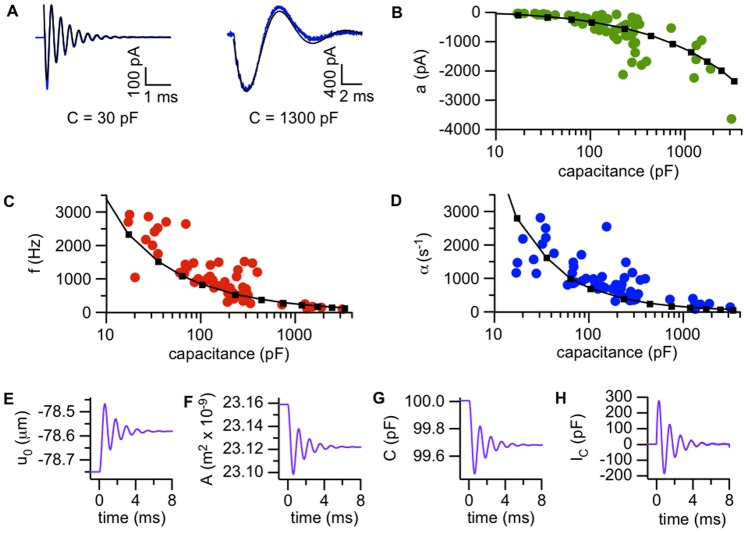
Bilayer tension and fluid dynamics determine the amplitude and time course of the response to ultrasound. **A.** Examples of ultrasound-induced currents (Off response) in low-capacitance (30 pF) and high-capacitance (1300 pF) bilayers. The blues lines are the average of 20–50 ultrasound applications and the black lines are fits of Eq. 1. Values of the fit parameters (± SD) are *a*, −290±5 pA; *f*, 2680±5 Hz; *α*, 1370±30 s^−1^; *φ*, −0.68±0.01 radians for the 30-pF bilayer; and *a*, −1570±10 pA; *f*, 150±0.3 Hz; *α*, 270±2 s^−1^; *φ*, 0.06±0.01 radians for the 1300-pF bilayer. Ultrasound was at 1 MHz and 610 mW/cm^2^, and the voltage-clamp potential was −200 mV. The baseline currents have been subtracted. **B–D.** Amplitude (***B***), frequency (***C***) and exponential damping constants (***D***) for the Off component of the capacitive current in response to ultrasound at 1 MHz and 610 mW/cm^2^, under voltage-clamp at −200 mV, as a function of bilayer capacitance (logarithmic scale) (*n* = 59). The small squares joined by black lines were obtained from solutions of Eq. 13 with various values of the bilayer diameter, with the solution density *ρ* = 1000 kg/m^3^, solution viscosity *η* = 1 mPa⋅s, bilayer tension *γ* = 0.8 mN/m, hydrostatic pressure *P_0_* =  −70 N/m^2^, and radiation-force pressure *P_US_* = 0.15 N/m^2^. **E–H.** Simulated changes in displacement (*u_0_*, measured at the center of the bilayer) (***E***), changes in area (***F***), changes in capacitance (***G***), and capacitive current (***H***) for the On component of ultrasound response in a 100-pF bilayer, obtained by solving Eq. 13 with a bilayer diameter of 120 µm and other parameters as in part ***D***. The time is relative to the start of the ultrasound application.

The amplitude and time course of the capacitive current response depend on bilayer capacitance (**Fig. 7**). Amplitude increases with capacitance (**Fig. 7** **** ***B***), while the frequency and damping constants both decrease with capacitance before approaching plateau values (**Fig. 7** **** ***C*** **and** ***D***). This suggests that in response to ultrasound, the bilayer oscillates at a specific resonant frequency determined by its diameter. Following work by Kramer [25], we modeled the dynamic response to radiation force as a transverse wave along the fluid interface defined by the bilayer, driven by bilayer interfacial tension (*γ*), hydrostatic pressure (*P_0_*), and the pressure due to radiation force (*P_US_*).

We determined the time course of the bilayer displacement *u*(x, t) (see **Fig. 5** ***A***) in response to ultrasound by solving a version of the Navier-Stokes equation of fluid dynamics, and used this time course to calculate the capacitive current response (see **Materials and Methods**, *“Model of the dynamic response to ultrasound”* for details). With a value of *γ* (0.8 mN/m) within the range previously reported for bilayers formed from decane solutions (∼0.4–6.5 mN/m) [38], [39], [40], [41], the model can reproduce the experimental relationship between bilayer capacitance and the response amplitude, frequency, and damping constant across three orders of magnitude (**Fig. 7** ***B–D***). Based on the scatter of the experimental data relative to the modeled curves, there appears to be considerable variability in the interfacial tension of bilayers formed from the same lipid solution. This variability has been remarked upon by other workers studying bilayer vibrations [39]. In addition, the experiments were conducted at room temperature with no particular effort to control the ambient temperature, so small day-to-day variations in temperature may also be a source of variability.

As the model indicates, the displacements underlying the capacitive currents are small. As an example, for a 100-pF bilayer the displacement in response to ultrasound at the center of the bilayer (where the displacement is greatest) is 0.17 µm at steady state, reaching a peak of 0.28 µm during the dynamic response (**Fig. 7** ***E***). The corresponding peak and steady-state area changes are 0.26 and 0.16% (**Fig. 7** ***F***).

The model provides an estimate of *P_US_* that is consistent with the radiation force hypothesis. The maximal pressure from radiation force that ultrasound can produce is 2*I/*c*, where *I* is the intensity and *c* is the speed of sound [21]. For ultrasound at 610 mW/cm^2^ in an aqueous solution, this gives a maximal pressure of 8.1 N/m^2^; the estimated value of *P_US_* is 0.15 N/m^2^, lower than this limiting value. In addition, the model explains the dependence of the response amplitude on the hydrostatic pressure (*P_0_*) (**Fig. 5** ***B*** **and** ***C***). For small values of *P_0_*, the bilayer is nearly flat in the resting state. When ultrasound is applied to a bilayer with relatively small curvature, the total pressure (*P_0_*+ *P_US_*) and opposing pressure due to the interfacial tension (2*γ*(δ^2^ *u*/δt^2^)) can be equalized with a relatively small displacement, resulting in a relatively small change in area. As the hydrostatic pressure and initial curvature increase, increasingly large displacements and area changes are necessary to offset the same radiation force per unit area.

### The Response to Ultrasound Depends on the Mechanical Properties of the Lipid Membrane

We conclude that the amplitude and time course of the response of lipid bilayers to ultrasound depend on the mechanical properties of the bilayer, and in particular on the interfacial tension. It should therefore be possible to modify the response by changing the lipid composition of the bilayer. In preliminary experiments on bilayers of different phospholipid composition, we observed oscillating capacitive currents in response to ultrasound with frequencies and damping constants similar to those seen with POPE/POPG bilayers (**Table S1**). However, we cannot reliably determine bilayer tension from these data (from 6–9 bilayers per lipid composition), since there is considerable variability in the ultrasound response among bilayers formed from the same lipid solution (**Fig. 7** **** ***B*** **–** ***D***), presumably reflecting variability in mechanical properties. Independent of this, the response also varies with bilayer diameter, which cannot be controlled precisely. Thus large data sets, incorporating bilayers spanning a wide range of diameters, are required to investigate bilayer mechanical properties with the approach used here. A detailed investigation of the dependence of the ultrasound response on lipid composition is therefore outside the scope of this article.

**Figure 8:**
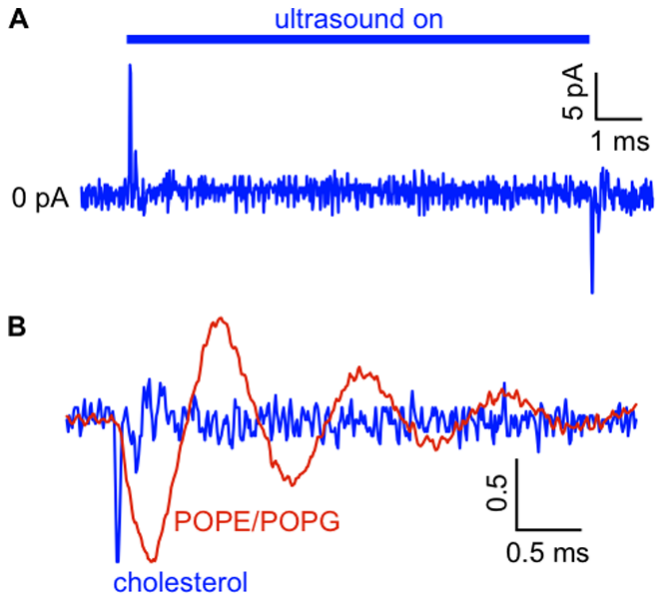
On/Off current in response to ultrasound in cholesterol membranes. **A.**. Current in response to a 10-ms ultrasound application at 1 MHz and 610 mW/cm^2^ in a cholesterol membrane voltage-clamped at −200 mV. The current trace is the average of 50 ultrasound applications. The capacitance of the membrane was 130 pF and the peak negative current is −11 pA. The mean (± SE) peak negative current was −18±3 pA (*n* = 10, capacitance range 70–130 pF). This small current response was not seen when the aperture on the bilayer partition was occluded with a drop of lipid/decane solution, indicating that it depends on the presence of a nanometer-scale lipid film. **B.** Off current responses for the cholesterol membrane in ***A*** (*blue current trace*) and in a POPE/POPG bilayer with the same capacitance in response to the same ultrasound stimulus (*red current trace*), normalized to the peak negative current. The current scale bar is in normalized units.

Nonetheless, we reasoned that it should be possible to resolve effects of lipid composition on the response to ultrasound using membranes with dramatically different mechanical properties than those formed by the phospholipids used in these experiments. Indeed, we found that membranes formed from cholesterol/decane solutions showed a response to ultrasound clearly different from that of phospholipid bilayers (**Fig. 8**). The symmetrical On/Off behavior and the switch in current polarity between the On and Off responses are still apparent for cholesterol/decane membranes, but the amplitude is much smaller and the time course of the response is quite different, consisting of a single, brief current spike. (There is possibly some indication of oscillation, but it is at or below the level of the background noise, and we were unable to consistently fit Eq. 1 to the current response in cholesterol membranes). This result is consistent with the effects of cholesterol on membrane mechanical properties. Cholesterol (40% by weight) was previously found to increase the interfacial tension of 1,2-dielaidoyl-sn-3-glycero-phosphocholine bilayers from 0.42 to 2.45 mN/m, and to increase interfacial viscosity (which is negligible in the absence of cholesterol) to 10 nPa⋅s [41]. Although similar data are not available for 100% cholesterol membranes, the increase in tension and interfacial viscosity is qualitatively consistent with the apparently higher frequency and much stronger damping that we observe in cholesterol membranes.

### Laser Doppler Vibrometry Confirms Bilayer Displacement in Response to Ultrasound

**Figure 9:**
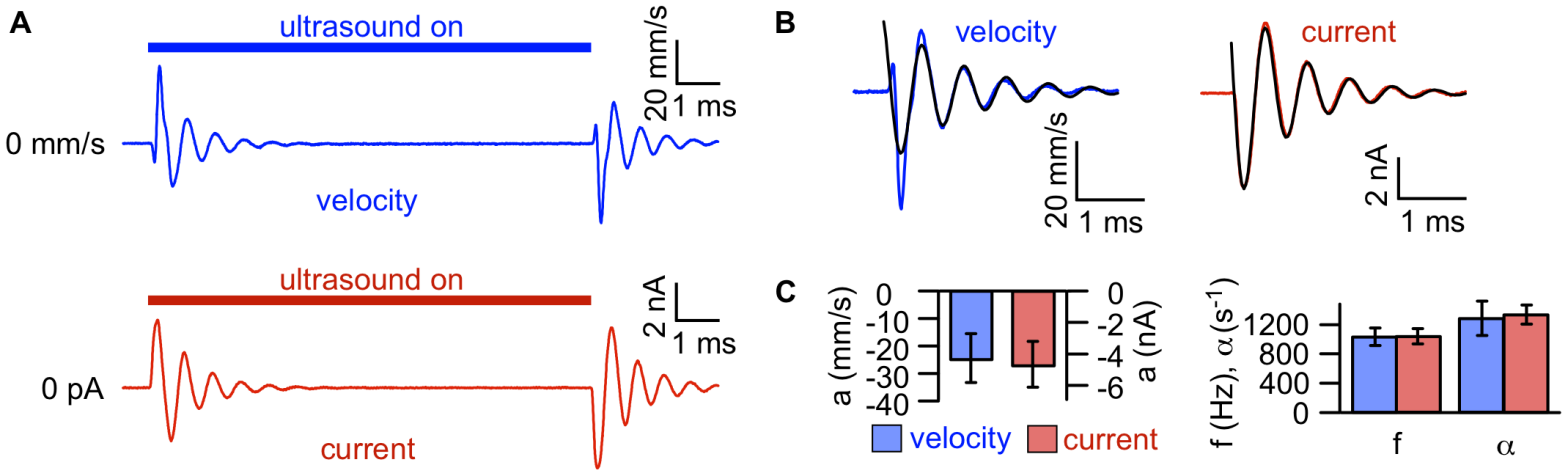
Similar time courses of ultrasound-induced capacitive currents and bilayer velocity measured by laser Doppler vibrometry. **A.** Bilayer velocity (*blue*) and current (*red*) in response to a 10-ms ultrasound application with a focused transducer at 43 MHz and 5 W/cm^2^, under voltage-clamp at −200 mV. The current and velocity traces are the average of 20 ultrasound applications. The capacitance of the bilayer was 380 pF. **B.** Fits of Eq. 1 (*black lines*) to the Off components of the velocity and current for the bilayer in ***A***. For velocity, values of the fit parameters (± SD) are *a,* −24±1 mm/s; *f*, 1600±5 Hz; *α*, 980±30 s^−1^; *φ*, −1.10±0.02 radians. For current, values of the fit parameters (± SD) are *a*, −5700±40 pA; *f*, 1600±2 Hz; *α*, 1200±10 s^−1^; *φ*, −0.42±0.01 radians. **C.** Mean (±SE) amplitude (*left*), and frequency (*f*) and damping constant (*α*) (*right*) from fits of Eq. 1 to the Off velocity (*blue*) and current (*red*) in response to ultrasound at 43 MHz and 5 W/cm^2^, under voltage-clamp at −200 mV.

The data so far suggest that in response to ultrasonic radiation force, bilayers undergo oscillating displacement parallel to the direction of ultrasound propagation before settling to a steady-state configuration with increased or decreased curvature (depending on whether the bilayer is curved toward or against the direction of ultrasound propagation). To confirm this idea, we used a laser Doppler vibrometer to measure the velocity of the solution above the bilayer while simultaneously measuring the capacitive current in response to ultrasound. We hypothesized that with a sufficiently thin layer of solution covering the bilayer, the motion of the bilayer is coupled to that of the solution/air interface above it, so that the velocity measured at the interface is representative of the velocity of the bilayer. We therefore decreased the thickness of the solution layer above the bilayer by removing solution from the upper compartment of the chamber until both the bilayer and solution/air interface above it were within the focal volume of the vibrometer laser (see **Materials and Methods**). As we anticipated, the time courses of the capacitive current and interfacial velocity in response to ultrasound are strikingly similar (**Fig. 9**). The precise correspondence between the capacitive current and the velocity is consistent with the model described above (**Fig. 5** **** ***A***, **Fig. 7**), providing additional evidence that lipid bilayers undergo displacement in response to radiation force.

### A Direct Effect of Ultrasound on the Bilayer

We also discovered that the amplitude of the capacitive current response increased when the thickness of the solution layer above the bilayer was decreased relative to the thickness under our standard experimental conditions. Along with the apparent coupling between the movement of the bilayer and the movement of the interface, this result indicates that at least part of the radiation force causing the bilayer displacement is due to the large difference in acoustic impedance (density times speed of sound) between the solution covering the bilayer and the air above it. This impedance mismatch results in reflection of the ultrasound wave, generating a large intensity gradient at the solution/air interface, causing the interface to rise, and forcing the solution (and bilayer) below to move along with it.

**Figure 10:**
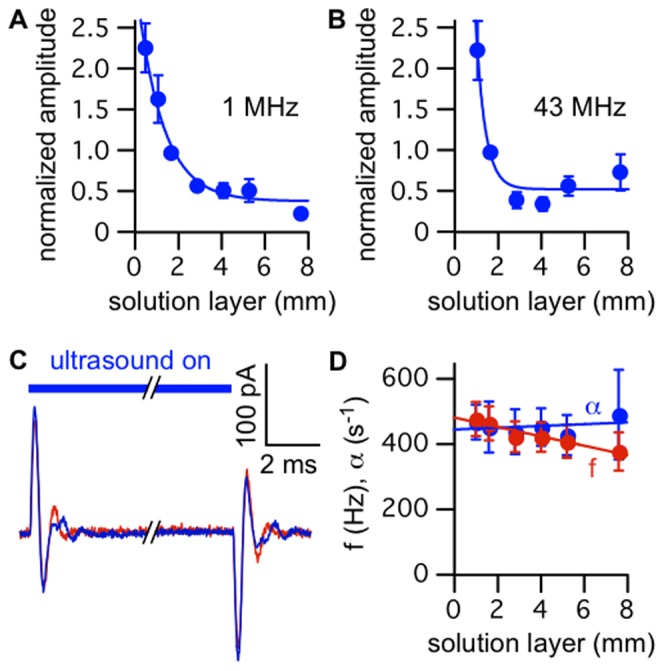
Part of the radiation force on the bilayer is due to a direct interaction with ultrasound. **A.** Mean (±SE) capacitive current response amplitude as a function of the thickness of the solution layer between the bilayer and the solution/air interface, normalized to the response amplitude at the reference thickness (1.7 mm, corresponding to the standard solution volume of 300 µL in the upper compartment of the chamber) and to the bilayer capacitance (see **Materials and** **Methods**, *“Data Analysis”* section), for ultrasound at 1 MHz and 610 mW/cm^2^ (*n* = 2–8). The solid blue line is an exponential fit with amplitude of 2.7±0.2, exponential decay constant of 0.84±0.14, and baseline value of 0.38±0.08 (parameters ± SD). **B.** As in ***A***, but for focused ultrasound at 43 MHz and 13 W/cm^2^ (intensity at focal spot, located on the bilayer) (*n* = 3–8). The solid blue line is an exponential fit with amplitude of 17±14, exponential decay constant of 2.3±0.8, and baseline value of 0.53±0.10 (parameters ± SD). **C.** Currents in response to ultrasound at 43 MHz and 13 W/cm^2^, under voltage-clamp at −100 mV, with an ∼8 mm thick solution layer between the bilayer and the solution air interface (*blue current trace*), and in response to the same ultrasound stimulus with the reflective solution/air interface replaced by an acoustic absorber (*red current trace*). The red current trace is the average of 10 ultrasound applications, and the blue current trace is the average of the currents before putting the acoustic absorber in place and after removing it, each the average of 10 ultrasound applications. The capacitance of the bilayer was 180 pF. **D.** Mean (±SE) capacitive current response frequency (*red circles*) and damping constant (*blue circles*) as a function of the thickness of the solution layer between the bilayer and the solution/air interface, for ultrasound at 1 MHz and 610 mW/cm^2^ or at 43 MHz and 13 W/cm^2^ (combined data sets from ***A*** and ***B***, *n* = 9–15). The solid lines are linear fits with slope and intercept of −14±2 Hz/mm and 480±9 Hz (frequency), and 2.7±4.3 s^−1^/mm and 440±20 s^−1^ (damping constant) (parameters ± SD).

Although at least part of the radiation force acting on the bilayer is due to the hydrodynamic coupling discussed above, there may also be other sources of radiation force. Also, interaction between the bilayer and the interface may in part determine the time course of the ultrasound response, contrary to our fluid dynamic model, which does not include this interaction. To clarify these issues, we investigated the relationship between the thickness of the solution layer above the bilayer and the response to ultrasound. We reasoned that by increasing the volume of solution in the upper compartment of the bilayer chamber, thereby increasing the thickness of the solution layer between the bilayer and the interface, we could eliminate the hydrodynamic coupling between them. In experiments with both the 1-MHz (**Fig. 10** **** ***A***) and 43-MHz transducers (**Fig. 10** **** ***B***), the amplitude of the capacitive current response decreased with increasing solution volume, confirming the importance of hydrodynamic coupling with the solution/air interface. However, the amplitude did not decline to zero as the solution level increased, but rather appeared to approach a non-zero plateau value (38% of the response at the reference position for the 1-MHz transducer and 53% for the 43-MHz transducer). This result indicates that a second source of radiation force acts on the bilayer, and that coupling of the bilayer movement with that of the solution/air interface is not necessary to produce a response to ultrasound.

Increasing the volume of solution in the upper compartment simultaneously changes several variables relevant to the capacitive current response: 1) increasing the thickness of the solution layer between the bilayer and the solution/air interface decreases the hydrodynamic coupling between them, tending to decrease the amplitude of the response; 2) increasing the distance between the transducer and the interface decreases the intensity of the ultrasound at the interface, due to spreading of the ultrasound beam, also tending to decrease the amplitude of the response (this accounts for the steeper decline of the response amplitude with increasing solution layer thickness for the focused 43-MHz transducer than for the 1-MHz transducer (**Fig. 10** **** ***A*** **and** ***B***)); 3) increasing the hydrostatic pressure on the bilayer increases its curvature and therefore increases its responsiveness to ultrasound, counteracting the tendency of the first two effects. Although it is difficult to separate the effects of these several variables, it is clear that some radiation force continues to act on the bilayer after the effect of the solution/air interface has been eliminated.

To confirm that the plateau component of the response is independent of the radiation force at the solution/air interface, we placed a piece of acoustically absorbing foam in contact with the solution in the upper compartment of the chamber after filling the compartment to its maximal volume (corresponding to an ∼8 mm thick solution layer). The presence of the acoustic absorber had no significant effect on the capacitive current response (**Fig. 10** ***C***), confirming that the interface does not interact with the bilayer when the solution layer between them is sufficiently thick, and that a second source of radiation force is responsible for the plateau component of the response. Thus in addition to the effect of hydrodynamic coupling with the solution/air interface, there is another other component of radiation force that apparently represents a direct effect of ultrasound on the bilayer.

We also note that the time course of the response with a solution layer sufficiently thick to eliminate the coupling between the solution/air interface and the bilayer is similar to that under our standard experimental conditions (where some coupling occurs). This can be seen by comparing the frequencies and damping constants obtained with a 7.6 mm thick solution layer (corresponding to the plateau region of the plots of thickness versus amplitude (**Fig. 10** ***A*** **and** ***B***)) with those obtained with a 1.7 mm (standard thickness) solution layer (**Fig. 10** ***D***). This result indicates that the solution/air interface does not play a major role in determining the time course of the response.

## Discussion

In contrast to earlier studies that found no effect of low-intensity ultrasound on the electrical properties of lipid bilayers [17], [18], our experiments clearly demonstrate dynamic and steady-state changes in bilayer capacitance in response to low-intensity ultrasound. The difference between the conclusions of these studies and ours may reflect differences in experimental design and bilayer properties. The authors of these studies looked for steady-state effects of ultrasound on capacitance by monitoring the current in response to a triangular voltage wave. It would therefore not be surprising if they failed to detect transient capacitive currents in response to the onset and offset of ultrasound, and small steady-state changes in capacitance may not have been detectable with this method. In fact, Rohr et al. [18] report a resolution of ±3% for their measurements of capacitance changes, which would have been insufficient to detect the small changes in capacitance that we observe (Pohl et al. [16] do not report a resolution but it was probably similar). Bilayer composition may also play a role in the apparent lack of a response to ultrasound in these earlier studies. Rohr et al. studied membranes formed from cholesterol in octane, while Pohl et al. used phosphatidylcholine and cholesterol in decane at a ratio of 2∶1 by weight. We find that membranes formed from cholesterol solutions are much less responsive to ultrasound (**Fig. 8**), which probably reflects a combination of higher tension and much higher interfacial viscosity [41].

The two possible sources for mechanical effects of ultrasound on biological tissue are radiation force and cavitation. In the model-membrane studies described here, we conclude that the effects of ultrasound are caused by radiation force. In our initial experiments, a large part of the radiation force was generated by reflection of ultrasound at the solution/air interface above the bilayer and coupled to the bilayer through the intervening solution. A similar phenomenon due to reflection of ultrasound at interfaces between tissues with a large impedance mismatch may be relevant to the effects of ultrasound *in vivo*. In addition to the radiation force generated at the solution/air interface, we found a second source of radiation force that may be intrinsic to the interaction of ultrasound with the bilayer (**Fig. 10**) and therefore also relevant to the effects of ultrasound on biological membranes.

We can use the results of **Fig. 10** ***A*** along with our model of the response to ultrasound (**Fig. 5** ***A*** **,** **Fig. 7**) to estimate the pressure resulting from this second source of radiation force. For the 1-MHz transducer, the amplitude of the plateau component of the response is 38% of the response under our standard experimental conditions (**Fig. 10** ***A***). Based on the dependence of the response parameters on bilayer capacitance (**Fig. 7** ***B–D***), we estimate that the radiation force per unit area for this transducer under our standard conditions is 0.15 N/m^2^. Thus, the radiation force per unit area for the plateau component is on the order of ∼0.05 N/m^2^.

There are two likely explanations for this additional radiation force. One explanation is that transmission of ultrasound across the bilayer involves a decrease in ultrasound intensity, due to either absorption or scattering of acoustic energy by the bilayer. This mechanism is intriguing since, as a fundamental interaction between acoustic waves and lipid membranes, it may also occur in the membranes of cells exposed to ultrasound. A second explanation for the additional radiation force is that it arises from an acoustic streaming process occurring in the bilayer chamber. Acoustic streaming is an effect of continuous intensity gradients in fluids, rather than discrete changes in intensity at acoustically mismatched interfaces. If ultrasound intensity in a fluid medium decreases in the direction of ultrasound propagation, the fluid within the ultrasound beam acquires a momentum in the direction of ultrasound propagation, with a compensating circulatory flow occurring outside the ultrasound beam [21], [22]. Acoustic streaming in response to ultrasound was previously reported to enhance the diffusion of acetic acid in the solution adjacent to a lipid bilayer [16], [17]. The intensity gradients driving acoustic streaming can result from absorption or, as most likely occurs in our experiments, from the intrinsic spatial profile of the ultrasound field. Both of these mechanisms may be relevant in biological tissues.

The mechanical properties of lipid bilayers have been shown to be sensitive to the phase state of the bilayer. Previously, it was demonstrated that the relaxation time of lipid vesicles in response to a pressure perturbation, as measured by thermal calorimetry, is proportional to the specific heat of the lipid membranes [42]. As a result, the temperature-dependence of the relaxation time shows a pronounced peak over the range of temperatures corresponding to the phase transition between the gel and fluid states of the lipids, paralleling the temperature-dependence of the specific heat. The compressibility of lipid monolayers shows a similar dependence on specific heat [43], as does the lifetime of transient pores in lipid bilayers [44]. We therefore anticipate a similar phase-state dependence in the response of lipid bilayers to ultrasound. In future experiments, it would be informative to determine to what extent the response we observe is dependent on the bilayer being in the vicinity of a phase transition. This may be relevant to the effects of ultrasound *in vivo* since biological membranes may be in the vicinity of phase transitions at physiological temperatures [45].

The question remains how the effects we observe in lipid bilayers are related to effects of ultrasound *in vivo*. Although radiation force is responsible for the effects of ultrasound on our system, it is not yet clear whether the effects of cavitation, radiation force, or both are involved in generating the effects of ultrasound on neural activity. It has been reported that ultrasound’s effects on neural activity in the mouse motor cortex *in vivo* are dependent on ultrasound frequency [7], [10], with the lowest frequencies tested being most effective, an observation more consistent with cavitation than radiation force. However, this result may reflect other effects of frequency, such as changes in beam profile, or decreased efficiency at higher frequencies due to greater absorption. In support of this latter interpretation, modulation of neural activity in the retina *in vitro* is effective at 43 MHz [6], and a response at this high frequency is strongly suggestive of a radiation force mechanism.

Certain aspects of our results are particularly intriguing in the context of ultrasonic neuromodulation. In practice, several protocols for neuromodulation imply that ultrasound is more efficient when delivered as a series of short (0.05–50 ms) applications [8], [9], [10], [11] than as a single long application with the same total exposure time (but see also reference 7). One group even found that ultrasound could either stimulate or inhibit neural activity depending on the duration of the application [11]. These facts suggest that dynamic effects associated with the onset of the ultrasound application may be important for *in vivo* neuromodulation, an interesting parallel with our results in which the peak bilayer displacement during the On response is approximately two thirds larger than that at steady-state (**Fig. 7** ***E***). It is also interesting that the magnitude of the bilayer displacement in our experiments depends on bilayer curvature (**Fig. 5** ***B*** **and** ***C***), since neurons contain specialized structures with highly curved membranes such as dendritic spines and synaptic vesicles, which are prime candidates for the locus of neuromodulatory effects.

The effects of radiation force that we observe in model membranes could translate to effects on neural activity *in vivo* through several different modalities, acting separately or in combination. One possibility is changes in ion channel activity due to membrane-protein interactions, such as those mediated by hydrophobic mismatch [46], [47] or membrane tension [47]. Another possibility is that some amplifying event converts small ultrasound-induced membrane displacements into functionally significant effects. For example, displacement of docked synaptic vesicles could cause them to fuse with the presynaptic membrane, resulting in neurotransmitter release, or a small displacement could cause intracellular membranes to fuse with the plasma membrane (as observed in response to pressure in mouse myocyte membrane patches [48]), depolarizing the membrane through an increase in capacitance [49]. As these possibilities indicate, our results in model membranes provide a foundation for further work investigating the effects of low-intensity ultrasound on more complex biological systems.

## Materials and Methods

### Lipid Bilayers

Phospholipid bilayers were formed from solutions of 1-palmitoyl-2-oleoyl-*sn*-glycero-3-phosphoethanolamine (POPE) and 1-palmitoyl-2-oleoyl-*sn*-glycero-3-phospho-(1′-*rac*-glycerol) (POPG) at a ratio of 3∶1 by weight. Except in **Fig. 4**, POPE/POPG bilayers were formed from 5 mg/mL solutions in decane. The bilayers in **Fig. 4** were formed from 8.3 mg/mL solutions in squalene. Cholesterol membranes were formed from 5 mg/mL solutions in decane. Lipids (Avanti Polar Lipids, labaster, AL) were stored at −80°C as chloroform solutions. After warming to room temperature, the lipids were evaporated to a film with argon, resuspended in pentane and dried with argon five times, and finally resuspended in decane or squalene at the working concentration.

Bilayers were formed on tapered apertures, ∼0.1–1 mm in diameter at the apex, in partitions horizontally separating two compartments filled with buffered salt solutions. The partitions were made from 3 M transparency film (∼0.2 mm thick) and the apertures were made using the corner of a razor blade. To form bilayers, 1 µL of lipid/decane solution was applied to the aperture and allowed to dry for at least 30 minutes before adding solutions to the chamber and dragging an air bubble coated with a thin film of lipid/decane solution across the hole with a 10 µL pipette tip. For lipid/squalene solutions the same procedure was followed but without the initial application of lipid solution. The recording solution was (in mM) 300 KCl, 10 Hepes, pH 7.0; or 500 CsCl, 10 Hepes, pH 7.0, in both the upper and lower compartments. The solution volume was 300 µL in each compartment, except where indicated otherwise. In experiments where the volume of solution in either compartment was changed, sufficient time (several seconds) elapsed for the bilayer and solvent border to equilibrate between adding/removing solution and applying ultrasound to the bilayer, as monitored by observing changes in bilayer capacitance. Bilayers were formed and experiments were conducted at room temperature.

### Electrophysiology

Voltage-clamp recording was performed using pClamp 9 software with a Digidata 1322A digitizer and either an Axopatch 200B or Multiclamp 700A amplifier, all from Axon Instruments (Molecular Devices, Sunnyvale, CA). Current-clamp recording was performed using the Multiclamp 700A with the feedback resistor set to 5 GΩ. To charge the membrane to the desired voltage, ∼2–3 pA of current was applied across the bilayer for a few seconds. The *RC* charging time constants for lipid bilayers are very long relative to the length of the ultrasound stimuli in our experiments, so there is essentially no charge transfer across the bilayer during the stimulus. Because of the long *RC* time constant, the voltage did not reach steady-state during the brief charging current, but we could control the voltage with accuracy of ±5 mV.

All recordings used are the average of at least ten trials. The sampling rate was 100 kHz and the cut-off frequency of the low-pass filter was 10 kHz. Capacitance was monitored by observing the current in response to voltage ramps between −50 and +50 mV at a rate of 1 mV/ms.

### Velocity Measurements

For velocity measurements we used a laser Doppler vibrometer (Polytec GmbH, Waldbronn, Germany) equipped with a high-frequency modular controller (model OFV-2700) and velocity output decoder (OVD-05) capable of measuring velocities in the 0.5 Hz to 10 kHz frequency range with a 60 mm/s peak-to-peak full scale providing a resolution of 0.3 µm/s in a 10 Hz bandwidth. The laser was a Class II HeNe operating at 633 nm and <1 mW output power, and the focal spot size was 15 µm. In these experiments, positive velocity is considered to be in the direction of ultrasound propagation. For these experiments, we measured the velocity in the vicinity of the bilayer based on the light reflected from the solution/air interface above the bilayer. To ensure that the movement of the solution/air interface is representative of the movement of the bilayer, the solution layer above the bilayer was made as thin as possible (<0.5 mm), such that the bilayer and the interface were both within the focal volume of the laser. To achieve this, we focused the vibrometer laser on the bilayer and then removed solution from the upper compartment of the chamber (starting volume 300 µL) until the intensity of the reflected light was maximal.

### Ultrasound

Transducers were mounted below the bilayer chamber on an *x*-*y*-*z* manipulator, and oriented perpendicular to the bilayer. The experimental apparatus is illustrated in **Fig. 1** ***A***. Ultrasound was generated using a nominally 0.25 inch (6.35 mm) diameter, 1-MHz, flat (i.e., unfocused) transducer (NDT Systems, Irvine, CA), or a custom-built, 43-MHz, focused transducer.

Voltage pulses exciting the transducers were produced using two function generators. The first function generator delivered sinusoidal voltage pulses at the transducer’s center frequency to the input of the power amplifier and was gated by a 5-V square pulse from the second function generator. The second function generator was triggered by a 5-V pulse from the digitizer. For the 1-MHz transducer, the first function generator was an Agilent 33220A (Agilent Instruments, Santa Clara, CA) and the output was amplified by an ENI 240L 50-dB power amplifier (ENI, Rochester, NY); for the 43-MHz transducer, the first function generator was an HP Model 8116A (Hewlett Packard, Palo Alto, CA) and the output was either amplified by an ENI 320L 50-dB power amplifier (ENI, Rochester, NY) or delivered directly to the transducer without amplification. The second function generator was an Agilent 33220A.

For experiments using the 1-MHz transducer, the bottom of the bilayer chamber was covered with a thin sheet of mylar sealed to the chamber with silicon grease. For experiments using the 43-MHz transducer, the chamber was sealed with a film of polyurethane or Saran Wrap held in place with a rubber O-ring. The transducers were coupled to the bottom of the chamber using a small column of distilled water or standard ultrasound coupling gel (Parker Laboratories, Fairfield, NJ).

For the 43-MHz transducer, the focal spot diameter is ∼90 µm, and the focal length is 4.3 mm. The focal spot was positioned using pulse-echo measurements of the reflection from the partition. The position along the *z*-axis was adjusted to maximize the echo signal. The position was then adjusted in the *x*-*y* plane while monitoring the echo signal, which was seen to disappear when the focus was moved onto the aperture. By gradually adjusting the position while monitoring the echo signal, the focus was positioned at the center of the aperture.

The beam from the 1-MHz transducer is collimated to ∼4 mm in diameter by a 4-mm orifice separating the upper and lower compartments. To align the 1-MHz transducer with the bilayer aperture, the aperture was centered in the field of view of a 10× magnification light microscope, and the bilayer chamber was then removed and the transducer was centered before replacing the chamber. The position of the transducer was then adjusted along the *x*, *y*, and *z* axes to maximize the response of the bilayer to ultrasound in voltage-clamp mode.

Ultrasound intensities are reported as the intensity during a single ultrasound application. The intensity for the 1-MHz transducer was determined from the pressure measured with a calibrated hydrophone (model HNP-0400, Onda Corporation, Sunnyvale, CA) according to
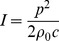
(6)where *p* is the peak acoustic pressure, *ρ_0_* is the density of the solution, *c* is the sound velocity (1500 m/s in water).

The intensity of the 43 MHz transducer was estimated by measuring the intensity at 20 MHz and correcting the measured intensity for the relative efficiency of the transducer at 43 MHz and 20 MHz. The intensity at 20 MHz was determined from the time-dependent displacement in response to ultrasound stimulation for an air/water interface positioned at the focus of the transducer. The displacement was measured using the laser Doppler vibrometer with a high frequency (50 kHz – 30 MHz) displacement decoder. The intensity was determined by:
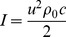
(7)where *u* is the peak velocity of the air/water interface. The frequency dependence of the transducer efficiency was determined based on two-way insertion loss measurements at a frequency range from 20 MHz to 50 MHz. Based on these measurements, the acoustic intensity at the focal spot is estimated as 5 or 13 W/cm^2^ for the experiments reported here.

### Data Analysis

Data were analyzed and models evaluated using Igor Pro (Wavemetrics, Lake Oswego, OR), Mathematica (Wolfram Research, Champaign, IL), or Microsoft Excel. The capacitive currents in response to ultrasound were generally stable over recording periods lasting several minutes to over an hour. The current has “On” and “Off” responses with usually very similar time courses (for example, **Fig. 1**). The On and Off currents always have a primary damped sinusoidal component, and occasionally have additional lower and higher frequency components. We did not attempt to analyze these additional components. We used the Off responses for further analysis, since these tended to show fewer irregularities, allowing for a more accurate determination of the parameters of Eq. 1.

In some experiments, we added solution to the bilayer chamber, which tends to increase bilayer capacitance by causing an increase in bilayer curvature, possibly accompanied by an increase in bilayer diameter. For the experiments in **Fig. 5**, we are interested in the effects of increased curvature at constant bilayer diameter. To compensate for changes in bilayer diameter, we normalized the current amplitude to capacitance using the plot of amplitude versus capacitance in **Fig. 7** **** ***B*** (for which the differences in bilayer capacitance are presumably due to differences in diameter). We normalized to capacitance using the equation
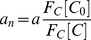
(8)Where *a* and *a_n_* are the raw and normalized current amplitudes, *C* and *C_0_* are the capacitance at the test volume and the reference volume, and the normalization function *F_C_* was obtained by fitting a power function to the plot in **Fig. 7** **** ***B***. For the experiments in **Fig. 10**, we are interested in the effects of solution volume on the sound field in the bilayer chamber, rather than the effects on the bilayer, so we also used this normalization procedure to compensate for the effects on bilayer diameter.

We determined the net change in bilayer capacitance in response to ultrasound by two methods. First, we integrated the right-hand side of Eq. 1 from *t* = 0 to *t* = ∞ with values of the parameters *a*, *f*, and *φ* determined from fits to the On capacitive current response, and divided the result by the voltage-clamp potential, as per Eq. 2. Second, we integrated the capacitive current directly after subtracting the baseline current, divided by the voltage-clamp potential to obtain the capacitance change as a function of time, and took the average of the capacitance in a 1-ms window at the end of the pulse. Both of these approaches generally gave similar results. However, integration of the capacitive current revealed a slow component to the capacitance change not immediately apparent in the capacitive current response. (Similarly, a small slow component can be seen in the voltage changes induced by capacitance changes in **Fig. 3** **** ***A***). We suspect that these slow components may represent a non-physiological process involving exchange of lipid molecules between the bilayer and the torus of lipid/decane solution around its perimeter. In contrast, we believe that the fast oscillating component of the capacitance changes represents a physiologically relevant elastic expansion and compression of the bilayer (see the discussion in connection with **Fig. 6** below). Since we are primarily interested in the fast, physiologically relevant capacitance changes, we use the first method (based on fitting Eq. 1 to the capacitive current) to calculate the net capacitance change in the analysis that follows.

For the analysis in **Fig. 6**, we need an estimate of the bilayer perimeter. The bilayers in our experiments are curved due to a hydrostatic pressure gradient (see **Fig. 5**), so the bilayer perimeter cannot be determined directly from measurements of the bilayer capacitance without additional information about the bilayer curvature. We therefore used the model in **Fig. 7** to estimate the bilayer perimeter. We fit a power function to the theoretical relationship between capacitance and perimeter, and used this to convert the measured bilayer capacitance to the perimeter. The bilayer perimeters estimated in this manner were consistent with the size of the apertures in the partitions as measured with an optical microscope.

### Model of the Dynamic Response to Ultrasound

To describe the dynamic response of lipid bilayers to ultrasound, we must account for the interactions between the bilayer and the surrounding solution so we are dealing with a problem in fluid dynamics, which requires a solution of the Navier-Stokes equation. For an incompressible fluid, the Navier-Stokes equation is
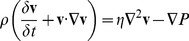
(9)where *ρ* is density, **v** is velocity, *η* is viscosity, and ∇*P* is the pressure gradient. To simplify the problem, we assume that the bilayer diameter is constant and perfectly circular and that bilayer displacements are only in the direction parallel to the direction of ultrasound propagation. We define our coordinate system so that the *x*-*y* plane coincides with the plane of zero bilayer curvature and the *z*-axis passes through the center of the bilayer. Thus, if the bilayer is perfectly flat, the center of the bilayer is located at the origin of the coordinate system (refer to **Fig. 5** **** ***A***). Since the fluid is incompressible and the displacements are only in the *z* direction, δ*v_z_*/δz = 0, so we can eliminate the nonlinear term **v**⋅∇**v**. In addition, δ*v_z_*/δx = d*v_z_*/δy due to the bilayer symmetry, so the equation becomes



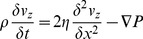
(10)To solve for the bilayer displacement we need an expression for the pressure gradient (∇*P*) at the point (x, y, *u_z_*) where *u_z_* is the bilayer displacement at (x, y). The pressure at (x, y, *u_z_*) is the sum of the external combined hydrostatic pressure (*P_0_*) and radiation-force pressure (*P_US_*), and the opposing pressure due to bilayer tension, *γ*(δ^2^ *u_z_*/δx^2^+ δ^2^ *u_z_*/δy^2^) = 2*γ*(δ^2^ *u_z_*/dx^2^). According to Kramer [25], the pressure decays with distance from the bilayer as
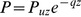
(11)where *P_uz_* is the pressure at z = *u_z_*, and *q* is the spatial frequency of the displacement, equal to 2π/*λ*, *λ* being the wavelength of the displacement. The condition of constant bilayer diameter imposes the boundary condition *u_z_*(−*d*/2, t) = *u_z_*(*d/2*, t) = 0 (where *d* is the bilayer diameter), so the displacement can be regarded as a standing wave with wavelength equal to twice the bilayer diameter. The pressure gradient at (x, y, *u_z_*) is therefore given by



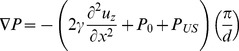
(12)Substituting this expression into Eq. 5 and dropping the subscript *z* gives the partial differential equation for the cross-sectional bilayer displacement *u*(x, t) (**Fig. 5** **** ***A***),
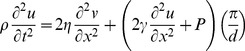
(13)where *ρ* is the density of the solution surrounding the bilayer, *η* is the viscosity of the solution, *v* is δ*u*/δt, *γ* is the bilayer interfacial tension, *P* is the pressure on the bilayer, and *d* is the bilayer diameter. During the ultrasound pulse, *P* is equal to the sum of the hydrostatic pressure (*P_0_*) and the radiation force per unit area (*P_US_*); otherwise *P* is equal to *P_0_*.

To model the dependence of the response to ultrasound on bilayer capacitance, we solved Eq. 13 numerically for bilayers of various diameters, with the boundary condition *u*(−*d*/2, t) = *u*(*d*/2, t) = 0, and the initial conditions *v*(x, 0) = 0 and the initial displacement *u*(x, 0) (due to the hydrostatic pressure) obtained by solving
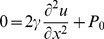
(14)with the boundary condition given above. Establishing the initial displacement in this manner is equivalent to allowing the bilayer to equilibrate with the solvent border at the hydrostatic pressure *P_0_*.

Partial differential equations were solved numerically with the method of lines using Mathematica’s built-in partial differential equation solving algorithm. Partial differential equations for the bilayer displacement *u*(x, t) were converted into a series of ordinary differential equations with respect to the variable *x*, which were discretized and solved using the standard built-in numerical ordinary differential equation solving algorithm. The bilayer area was determined from the integral
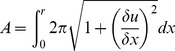
(15)


Numerical integration was performed using the built-in adaptive quasi-Monte Carlo algorithm. Changes in bilayer thickness were determined from the changes bilayer area according to Eq. 5 with *L_0_* = 4 nm (typical of bilayers containing solvent molecules [26], [27]). Capacitance as a function of time was determined from the simulated time course of *L* and *A* using Eq. 4 with *ε* = 2. Simulated capacitive currents were calculated using Eq. 2 and sampled at 100 kHz and analyzed in the same manner as the experimental data. The parameters *γ*, *P_0_*, and *P_US_* were manually adjusted to obtain a good fit to the experimental data; the values presented are not the result of an iterative fitting algorithm.

A few simplifications involved in the model are worth mentioning. First, we regard the bilayer as having a fixed diameter. In other words, the point of contact between the bilayer and the solvent border is fixed in space. In reality, the solvent border has some viscoelasticity [28] and a more complicated boundary condition would be necessary to account for this fact. We also do not include small changes in bilayer tension that may occur as the bilayer is stretched and compressed. Undoubtedly, the fluid flow in the chamber is more complex than that described in the Navier-Stokes model, and the assumption of zero velocity gradients cannot hold throughout the chamber. (However, the results in **Fig. 9** indicate that the fluid immediately above the bilayer does indeed move with a velocity similar to that of the bilayer, so this assumption appears to be appropriate in the vicinity of the bilayer). Finally, it is obviously unlikely that bilayers have a perfectly circular perimeter. We are nonetheless able to reproduce the relationship between the capacitive current response and the bilayer capacitance over three orders of magnitude using the accepted values for the density and viscosity of water at room temperature and a reasonable value for the bilayer tension (**Fig.** **7** **** ***B–D***). It therefore appears that these simplifications are not critical, particularly for the small-amplitude displacements modeled here.

## Supporting Information

Figure S1 **Response of lipid bilayers to ultrasound applications with modulated intensity predicted using a linear system model. A.** Step (*left*) and impulse (*right*) responses for ultrasound applied to a POPE/POPG bilayer voltage-clamped at −200 mV with ultrasound at 1 MHz and 610 mW/cm^2^. The capacitance of the bilayer was 270 pF. The On response to a standard ultrasound pulse is assumed to be the step response of the system, and the impulse response is obtained by differentiating the step response. The step response is the average of 20 ultrasound applications. The impulse response can be convolved with any ultrasound intensity profile to obtain the output current, as shown in ***B*** and ***C***. **B.** On and Off current responses (*blue current traces*) for the bilayer in ***A*** in response to ultrasound at 1 MHz with a quadratically rising and falling intensity profile (2 ms rise and decay times, steady-state intensity 610 mW/cm^2^), along with the predicted responses derived from the impulse response shown in ***A*** (*red lines*). **C.** As in ***B***, except the rise and decay times of the ultrasound intensity are 5 ms. **D.** Mean (±SE) peak positive (*top*) and negative (*bottom*) currents for the On (*solid circles*, *left*) and Off (*open circles*, *right*) responses to pulses with quadratically rising and falling intensities with rise and decay times of 0.5, 1, 2, and 5 ms, normalized to the peak currents for a step-like (unmodulated) ultrasound application (*blue lines and circles*), along with the mean normalized peak currents predicted using the corresponding step response for each bilayer (*red lines and circles*) (*n* = 7). The currents for a step change in intensity are designated as having zero rise time. The predicted values are for simulated currents generated by convolving the time course of the ultrasound intensity with the impulse response derived from the On response for each bilayer and adding Gaussian noise with standard deviation equal to that of the baseline noise in the corresponding current record. Some error bars are smaller than the symbol size.
(TIFF)

Table S1 **Amplitude and time course of the capacitive current in response to ultrasound in phosphocholine bilayers with different acyl chain groups.** Mean (± SE) values at −200 mV for the Off response to ultrasound at 1 MHz and 610 mW/cm^2^. Bilayers were formed from 5 mg/mL solutions in decane. Abbreviations: DOPC, 1,2-dioleoyl-*sn*-glycero-3-phosphocholine; DPhPC, 1,2-diphytanoyl-*sn*-glycero-3-phosphocholine; (20∶1)PC, 1,2-dieicosenoyl-*sn*-glycero-3-phosphocholine.
(DOCX)

File S1 **The response of lipid bilayers to ultrasound is a step response.** This supporting text, along with **Fig. S1**, demonstrates that the response of bilayers to ultrasonic radiation force is the step response of a linear system.
(DOCX)
