# Supplementary figures and images for: Dynamic Response of Model Lipid Membranes to Ultrasonic Radiation Force
Source: PLoS One. 2013 Oct 23;8(10):e77115. doi: 10.1371/journal.pone.0077115 (PMC3806737; doi:10.1371/journal.pone.0077115)

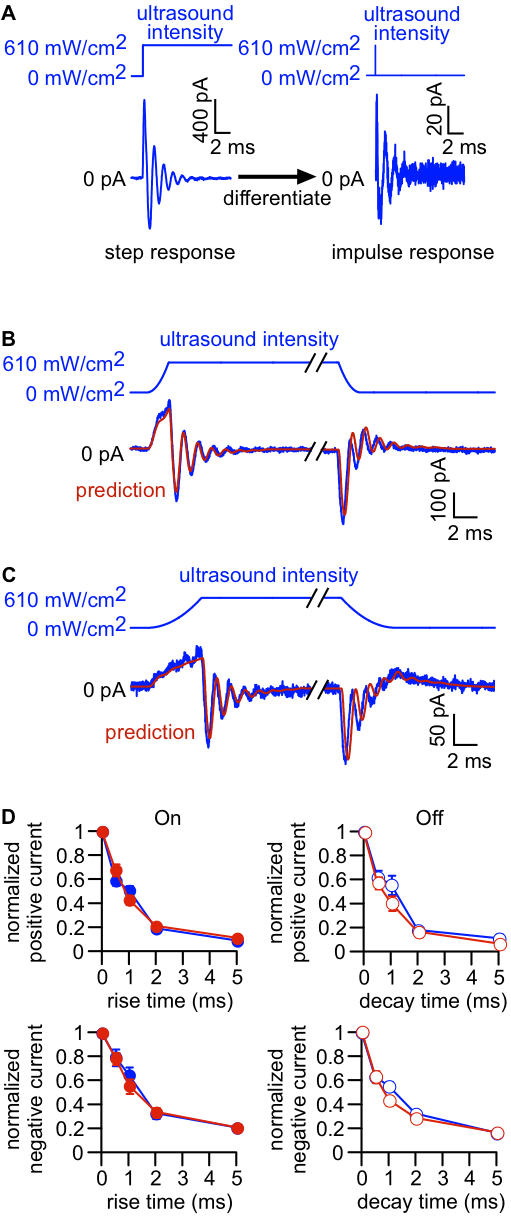

Supplement: Figure S1 — Response of lipid bilayers to ultrasound applications with modulated intensity predicted using a linear system model. A. Step (left) and impulse (right) responses for ultrasound applied to a POPE/POPG bilayer voltage-clamped at −200 mV with ultrasound at 1 MHz and 610 mW/cm2. The capacitance of the bilayer was 270 pF. The On response to a standard ultrasound pulse is assumed to be the step response of the system, and the impulse response is obtained by differentiating the step response. The step response is the average of 20 ultrasound applications. The impulse response can be convolved with any ultrasound intensity profile to obtain the output current, as shown in B and C. B. On and Off current responses (blue current traces) for the bilayer in A in response to ultrasound at 1 MHz with a quadratically rising and falling intensity profile (2 ms rise and decay times, steady-state intensity 610 mW/cm2), along with the predicted responses derived from the impulse response shown in A (red lines). C. As in B, except the rise and decay times of the ultrasound intensity are 5 ms. D. Mean (±SE) peak positive (top) and negative (bottom) currents for the On (solid circles, left) and Off (open circles, right) responses to pulses with quadratically rising and falling intensities with rise and decay times of 0.5, 1, 2, and 5 ms, normalized to the peak currents for a step-like (unmodulated) ultrasound application (blue lines and circles), along with the mean normalized peak currents predicted using the corresponding step response for each bilayer (red lines and circles) (n = 7). The currents for a step change in intensity are designated as having zero rise time. The predicted values are for simulated currents generated by convolving the time course of the ultrasound intensity with the impulse response derived from the On response for each bilayer and adding Gaussian noise with standard deviation equal to that of the baseline noise in the corresponding current record. Some error b [file pone.0077115.s001.tiff]
