# Supplementary material for: Dynamic Response of Model Lipid Membranes to Ultrasonic Radiation Force
Source: PLoS One. 2013 Oct 23;8(10):e77115. doi: 10.1371/journal.pone.0077115 (PMC3806737; doi:10.1371/journal.pone.0077115)
Supplement: Table S1 — Amplitude and time course of the capacitive current in response to ultrasound in phosphocholine bilayers with different acyl chain groups. Mean (± SE) values at −200 mV for the Off response to ultrasound at 1 MHz and 610 mW/cm2. Bilayers were formed from 5 mg/mL solutions in decane. Abbreviations: DOPC, 1,2-dioleoyl-sn-glycero-3-phosphocholine; DPhPC, 1,2-diphytanoyl-sn-glycero-3-phosphocholine; (20∶1)PC, 1,2-dieicosenoyl-sn-glycero-3-phosphocholine. (DOCX) [file pone.0077115.s002.docx]

|  | *a* (pF) | *f* (Hz) | *α* (s^-1^) | *capacitance range* (pF) | *N* |
| --- | --- | --- | --- | --- | --- |
| **DOPC** | -90 ± 20 | 530 ± 66 | 730 ± 63 | 64 - 300 | 9 |
| **DPhPC** | -150 ± 13 | 1100 ± 230 | 1000 ± 110 | 57 -150 | 6 |
| **(20:1)PC** | -75 ± 23 | 430 ± 28 | 440 ± 39 | 71 -230 | 7 |
