# Supplementary material for: Dynamic Response of Model Lipid Membranes to Ultrasonic Radiation Force
Source: PLoS One. 2013 Oct 23;8(10):e77115. doi: 10.1371/journal.pone.0077115 (PMC3806737; doi:10.1371/journal.pone.0077115)
Supplement: File S1 — The response of lipid bilayers to ultrasound is a step response. This supporting text, along with Fig. S1, demonstrates that the response of bilayers to ultrasonic radiation force is the step response of a linear system. (DOCX) [file pone.0077115.s003.docx]

**SUPPORTING INFORMATION.**

***The response of lipid bilayers to ultrasound is a step response*.**

The time course of the changes in bilayer area in response to ultrasound (**Fig. 7 *F***) resembles the response to a unit step function in many systems. This observation suggests that the time course of the radiation-force pressure applied to the bilayer is essentially an instantaneous step from zero to *P_US_*, as would indeed be expected based on the rise time of the ultrasound intensity (~5 μs for the 1-MHz transducer and ~50 ns for the 43-MHz transducer, faster than the 10 ms sample interval of our experiments and much faster than the apparent response time of the bilayer).

To confirm that the time course of the applied pressure is indeed a step function, we demonstrate below that, starting with the assumption that the response of the bilayer to an ultrasound application is the step response of a linear system, we can accurately predict the response to stimuli in which the ultrasound intensity is modulated by an envelope function. Based on this assumption, we can differentiate the On response to a standard ultrasound application (a step-like change in ultrasound intensity) to obtain the impulse response of the system (**Fig. S1 *A***), and then convolve the impulse response with any input to obtain the output. We verified this by modulating the sinusoidal voltage driving the ultrasound transducer with a voltage ramp, such that the intensity rises and falls quadratically (intensity is proportional to the square of the applied voltage) rather than in step-like fashion (**Fig. S1 *B*-*D***). The capacitive currents in response to modulated pulses with 2-ms (**Fig. S1 *B***) and 5-ms (**Fig. S1 *C***) rise times and final intensities of 610 mW/cm^2^ are shown in blue along with the predicted responses in red. The success of these predictions based on a simple linear system theory indicates that the time course of the pressure on the bilayer due to acoustic radiation force (for an unmodulated ultrasound application) is indeed a step function.
